# Cancer risks in first-degree relatives of CHEK2 mutation carriers: effects of mutation type and cancer site in proband

**DOI:** 10.1038/sj.bjc.6605038

**Published:** 2009-04-28

**Authors:** J Gronwald, C Cybulski, W Piesiak, J Suchy, T Huzarski, T Byrski, B Gorski, T Debniak, M Szwiec, D Wokolowczyk, M Matuszewski, P Sun, J Lubinski, S A Narod

**Affiliations:** 1 Department of Genetics and Pathology; International Hereditary Cancer Center, Pomeranian Medical University; Szczecin; Poland; 2 Oncology Regional Hospital; Opole; Poland; 3 Department of Urology; Medical University of Gdansk; Gdansk; Poland; 4 Women's College Research Institute; University of Toronto and Women's College Hospital, M5G 1N8; Toronto, Ontario; Canada

**Keywords:** CHEK2, breast cancer, colon cancer, prostate cancer

## Abstract

It is important to have accurate knowledge of the range of cancers associated with various CHEK2 mutations, and of the lifetime risks of cancer associated with each. We wished to establish the relationship between family history, mutation type and cancer risk in families with a CHEK2 mutation. We obtained a blood sample and pedigree information from 2012 unselected women with breast cancer, from 2007 men with prostate cancer and from 1934 patients with colon cancer, from hospitals throughout Poland. Genetic testing was carried out for four founder CHEK2 mutations on all 5953 specimens and 533 carriers were identified. We estimated the risk to age 75 for any cancer in the 2544 first-degree relatives to be 22.3%. After adjusting for mutation type, the risk of breast cancer was much higher among relatives of probands with breast cancer than among relatives of patients with prostate or colon cancer (HR=3.6; 95% CI=2.1–6.2; *P*=0.0001). Similarly, the risk of prostate cancer was higher among relatives of probands with prostate cancer than among relatives of patients with breast or colon cancer (HR=4.4; 95% CI=2.2–8.7; *P*=0.0001) and the risk of colon cancer was higher among relatives of probands with colon cancer than among relatives of patients with prostate or breast cancer (HR=4.2; 95% CI=2.4–7.8; *P*=0.0001). These analyses suggest that the risk of cancer in a carrier of a CHEK2 mutation is dependent on the family history of cancer.

In Poland, there are four founder mutations in CHEK2, including three truncating mutations (1100delC, IVS2+1G>A, del5395) and one missense mutation (I157T; Cybulski *et al*, 2004a, 2007a). CHEK2 mutations predispose men and women to a range of cancer types, including breast, prostate and colon (Cybulski *et al*, 2004b; Cybulski *et al*, 2007b). For breast and prostate cancer, the truncating mutations are associated with higher penetrance than the missense mutation (Cybulski *et al*, 2007c). For colon cancer, only the missense variant I157T is associated with an elevated risk (Cybulski *et al*, 2007b). The odds ratios reported to be associated with truncating and missense mutations for breast, prostate and colon cancer in our most recent studies are presented in Table 1.

Screening for CHEK2 mutations has not been well integrated into genetic counselling for cancer (Narod and Lynch, 2007). To some extent, this reflects the rarity of CHEK2 mutations and the differences in the prevalence of mutations from country to country. Only one mutation, 1100delC, appears to be widely disseminated (CHEK2 Breast Cancer Case-Control Consortium, 2004; Weischer *et al*, 2008; Zhang *et al*, 2008). Uncertainty remains as to the best estimate of cancer risk for gene carriers, and the factors which influence the penetrance of the *CHEK2* gene. Different methods have been used to measure penetrance; but it is generally accepted that studies based on series of unselected patients are superior to those based on cases selected for family history (Begg, 2002; Risch and Narod, 2003). If there are risk factors, which cluster within families, and which predispose to a cancer at a particular site, then we expect the risk of cancer in close relatives to depend on whether the proband was affected with the same type of cancer or with a different type.

We conducted population surveys on unselected cases of breast cancer, prostate cancer and colon cancer. Poland is well suited for association studies because the population is genetically homogenous and because of high patient participation rates. The patients underwent genetic testing for the four founder CHEK2 mutations. Patients with mutations were questioned regarding details about all cancers in their first-degree relatives. Using this data, we are able to estimate the cumulative incidence of all cancers in the first-degree relatives of the carriers, and to compare the risks by specific CHEK2 mutation, and by the disease status of the index case (breast or prostate or colon cancer).

## Materials and methods

### Study subjects

In the course of a national breast cancer survey, we interviewed 2012 women diagnosed with breast cancer at one of the seven centres situated throughout Poland from 2002 to 2003.

A total of 1934 patients with colon cancer were interviewed from 1998 to 2007 at 11 centres situated throughout Poland. The first 1050 colon cancer patients have been described in detail previously (Cybulski *et al*, 2007b). The study has since been extended to include more patients using the same methodology. In all, 2007 patients with prostate cancer were interviewed from 1999 to 2007 at 13 centres situated throughout Poland. The cancer patients have been described in detail previously (Cybulski *et al*, 2004b; Cybulski *et al*, 2007c). The study was approved by the ethics board of the Pomeranian Medical University.

### Laboratory analyses

A DNA sample was obtained from peripheral blood from each patient. These samples were assayed for four founder mutations in *CHEK2* gene (1100delC, IVS2+1G>A, del5395 and I157T). Methods have been described elsewhere (Cybulski *et al*, 2004a, 2007a).

### Pedigree data

The patient (index case with mutation) was interviewed in person or on the telephone by a member of the research team. He or she was asked to provide complete information about cancers in all first-degree relatives, including type of cancer, age of onset, age of death and current age of relatives without cancer. For unaffected relatives, age of death was recorded (if deceased) or current age (if alive). The diagnoses of cancer in the relatives were not confirmed by reference to pathology reports. Patients were unaware of their mutation status at the time of interview. Because of ambiguity expressed by probands in distinguishing ovarian, cervical and uterine cancer in relatives, these three sites were merged (female genital tract).

### Statistical analysis

We estimated the cancer risk for first-degree relatives of mutation carriers for all mutations and for each mutation separately, using Kaplan–Meier survival analysis. Subjects were considered to be at risk of cancer from birth until either the development of cancer; death from another cause; the date of patient interview or until the age of 75 years. Cumulative incidence curves were computed separately for each site of cancer and for all cancers combined (any cancer). Risks were computed separately for truncating *versus* missense CHEK2 mutations. Risks were computed for men and for women and for both sexes combined. Risks were compared for relatives of patients with cancer at the same site, and for cancers at different sites. To establish which of these factors were relevant for predicting the lifetime risk of cancer of each type, a Cox proportional hazards model was used which incorporated both the mutation type (missense *versus* truncating) and the site of disease in the proband (breast or colon or prostate).

## Results

Among the 5953 unselected Polish individuals with cancer in this study, 533 CHEK2 mutation carriers were identified, including 186 probands with breast cancer (9.2% of the total tested) (mean age of diagnosis: 55.6 years; range: 27–82 years), 147 probands with colon cancer (7.6% of total; mean age of diagnosis: 61.9 years; range: 25–88 years) and 200 probands with prostate cancer (10.0% of total; mean age of diagnosis: 65.7 years; range: 45–88 years). A total of 515 (96.6%) pedigrees contained sufficient data for statistical analysis. In total, these 515 pedigrees recorded 2544 first-degree relatives (1270 men and 1274 women). In all, 431 cancers were reported in the 2544 relatives (213 in men and 218 in women; Table 2). Data were missing for four subjects and these were excluded, leaving 2540 first-degree relatives for analysis.

A total of 15.5% of the female relatives and 14.3% of the male relatives have been diagnosed with some type of cancer by the age of 75. The cumulative risks of cancer at any of these sites among the first-degree relatives of the CHEK2 mutation carriers are shown in Table 3. For example, the risk of breast cancer to age 50 for first-degree relatives of breast cancer patients with the missense mutation was 3.2%, compared to a risk of 1.4% for women who were first-degree relatives of all individuals with this mutation. The risk of breast cancer for all female first-degree relatives of all truncating mutation carriers was estimated to be 7.6% to age 75, and of relatives of the missense mutations was 5.3%. The risk of prostate cancer for all first-degree relatives of all truncating mutation carriers was estimated to be 6.1% to age 75, and of missense mutations was 7.2%. The risk of colon cancer for all first-degree relatives of all truncating mutation carriers was estimated to be 2.0% to age 75, and of missense mutations was 3.5%.

**Figure 1:**
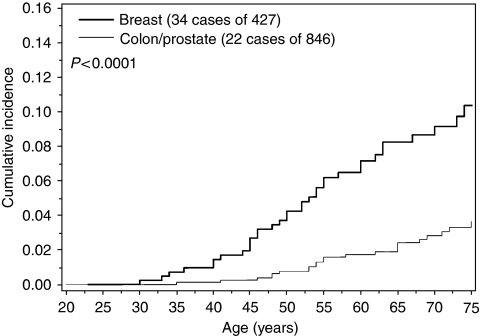
Cumulative incidence of breast cancer among first-degree female relatives of CHEK2 mutation carriers, by site of cancer in proband.

**Figure 2:**
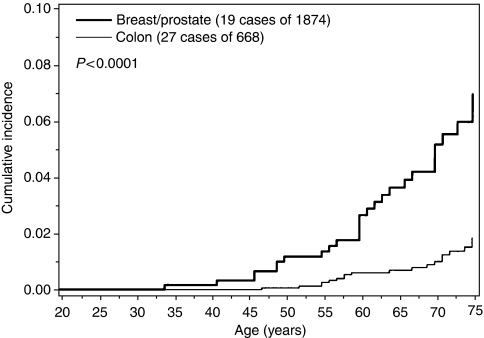
Cumulative incidence of prostate cancer among first-degree male relatives of CHEK2 mutation carriers, by site of cancer in proband.

**Figure 3:**
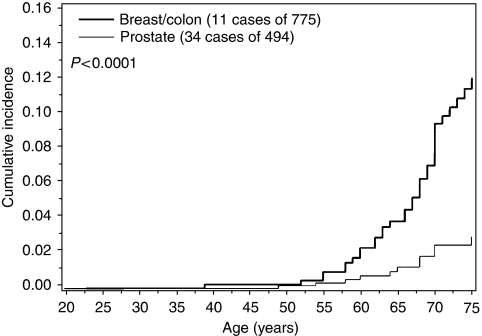
Cumulative incidence of colon cancer among first-degree relatives of CHEK2 mutation carriers, by site of cancer in proband.

The lifetime risk of cancer was higher for a specific cancer type when that type of cancer was also diagnosed in the proband (compared to a different site in the proband). The risk to age 75 for breast cancer among relatives of women with breast cancer was 10.4% compared to a 3.6% lifetime breast cancer risk among relatives of patients with colon or prostate cancer (Figure 1). The risk to age 75 for colon cancer among relatives of individuals with colon cancer was 7.0%, compared to a 1.9% lifetime colon cancer risk among relatives of patients with breast or prostate cancer (Figure 2). The risk to age 75 for prostate cancer among relatives of men with prostate cancer was 12.1%, compared to a 3.0% lifetime prostate cancer risk among relatives of patients with colon or breast cancer (Figure 3).

We constructed a Cox proportional hazard model to express the cancer risks for first-degree relatives of patients with mutations. A separate model was constructed for predicting the risks of breast, colon and prostate cancer in the first-degree relatives. For each model, the data were analysed with respect to the type of cancer diagnosed in the proband (breast, colonm and prostate) and the class of mutation (truncating *versus* missense). The relatives of breast cancer patients experienced a greater risk of breast cancer than did the relatives of the patients with other cancers (Table 4). A survival analysis using Cox regression estimated that the relatives of prostate/colon cancer patients experienced only approximately one-fourth the risk of breast cancer as did the relatives of breast cancer patients (Table 4). Similar results were obtained for prostate and colon cancer (Tables 5 and 6).

## Discussion

CHEK2 mutations confer substantial risks for breast, prostate and colon cancer, but the cancer risk appears to vary between and within populations. Potential sources of variation include different risks associated with different mutations, modifying genetic background and environmental or lifestyle factors. Ours is the first study to show that, among relatives of CHEK2 mutation carriers, the risks of breast, prostate and colon cancer differ dramatically depending on the type of cancer diagnosed in the proband. This supports the importance of the contribution of genetic background to risk modification in hereditary cancer syndromes (Byrnes *et al*, 2008). Environmental and lifestyle factors also play important roles, but these were not the topic of the current study. Our observation of a high degree of concordance in the clinical expression of cancer within a family is most readily explained by the existence of one or more modifying genes. Our data imply that the genetic modifiers are different for the three sites. For a given site, the underlying model might involve two genes, or represent a polygenic effect. In the simplest manifestation of a two-gene model, it would be necessary for a carrier of a deleterious CHEK2 mutation also to carry a specific allele of a second gene to develop cancer. If so, then all affected carriers would carry both alleles. The probability of a sibling inheriting both alleles would be 25%. Three of four siblings would not be at elevated risk for the index cancer and we would observe poor segregation in families between cancer and the presence of CHEK2 mutation.

The risks of breast cancer in first-degree relatives differed significantly, depending on whether the proband was diagnosed with breast cancer or another type of cancer. Our data support the model proposed by Byrnes *et al* (2008), whereby the risks associated with the cancer family history and with the genetic mutation act in combination (and are possibly multiplicative).

We have reported a similar finding among BRCA1 mutation carriers identified through unselected cases of breast or ovarian cancer (Warner *et al*, 1999; Moslehi *et al*, 2000; Gronwald *et al*, 2006) that is, among BRCA1 carriers, the risk of breast cancer was dependent on whether the affected relative had breast or ovarian cancer. The risk of ovarian cancer, however, was similar in the two groups of relatives (Warner *et al*, 1999; Moslehi *et al*, 2000; Gronwald *et al*, 2006). This suggests that among BRCA1 carriers, there are familial genetic modifiers for breast, but not for ovarian cancer. However, the familial modifying effects for CHEK2 on the risks of breast, colon and prostate cancer observed here were more extreme than those seen in our earlier studies of BRCA1 carriers.

Both truncating and missense CHEK2 mutations predispose to prostate cancer (Cybulski *et al*, 2007c). One modifying gene has been proposed for prostate cancer; using this database, we have recently shown that the risk of prostate cancer depends on the genotype of p27 (Cybulski *et al*, 2007c). The excess risk of prostate cancer attributable to a CHEK2 mutation was restricted to carriers of the VV genotype at p27. A similar effect was seen with the p27 genotype and the risk of colon cancer (Cybulski *et al*, 2007c). However, the p27 genotype did not modify the risk of breast cancer among CHEK2 carriers. We have recently shown that, among carriers of CHEK2 mutations, the risk of breast cancer was much greater for women who also carried a specific missense variant in BRCA2 and the interaction was statistically significant (*P*=0.03; Serrano-Fernández *et al*, 2008). BRCA2 is the first CHEK2-modifying gene proposed for breast cancer; however, the relevant BRCA2 allele (T1915M) was rare and was present in only 6% of the population controls and in 11% of the cases of CHEK2-associated breast cancer. It is likely that there are other risk-modifying genes and innovative strategies will be required to find these.

There are several strengths of our study. Ours is the only large single-centre study of unselected cancer patients originating in a well-defined, ethnically homogeneous group from one country to address the genetic epidemiology of CHEK2. Few patients declined to participate when approached. Subjects were unaware of their genetic status at the time the family history was obtained and all patient groups were studied using the same methods. Nevertheless, some of our individual risk estimates are imprecise and it will be important to continue to accrue patients to this and similar studies and to re-examine these questions in the future.

There are several limitations to our study as well. Despite the fact that we included a total of 5953 cancer patients, there were only 533 CHEK2-positive cases and 431 affected relatives. Many of our subgroup comparisons were based on small numbers; in particular, we had limited power to compare the risks associated with the different mutations for the three different sites. Our end point was the presence of cancer in first-degree relatives, and we did not have genotype status on the relatives. Ideally, we would compare cancer risks in relatives with and without CHEK2 mutations, but the requirement of collecting a blood or tissue sample from all first-degree relatives, living and dead, was prohibitive. Cancer diagnoses in relatives were based on proband recollection and were not confirmed with medical records. The lifetime risks of the specific cancer types appear to be low, and to some extent, this might represent incomplete reporting on the part of the proband; but the incidence rates for these three cancers are much lower in Poland than in the United States, and screening for these three sites is not widespread in the country. We did not have information on environmental, medical or lifestyle factors in the relatives. Finally, we did not have a control group of relatives of unselected, unaffected probands to measure cancer risk; we were concerned that healthy controls with a family history of cancer might be more willing than others to participate in a genetic epidemiology study of cancer and that if we included self-selected controls, we might generate biased risks.

The finding of phenotypic heterogeneity among CHEK2 carriers with the same mutation has potentially important clinical implications. Genetic counsellors should not provide risks simply based on the mutation status alone, and must incorporate the family history into the assessment as well. It is not clear to what extent the risks for breast, colon or prostate cancer are elevated in the cohorts of relatives with no family history of cancer at that site. It is also true that if a woman is found to be a CHEK2 carrier and has a family history of breast cancer, she is likely to be at high risk of breast cancer (Byrnes *et al*, 2008). Given that 70–80% of CHEK2-associated breast cancers are ER-negative, women with a mutation may be a good candidates for tamoxifen chemoprevention (Cybulski *et al*, 2009). Similarly, CHEK2 carriers with a family history of colon or prostate cancer may be good candidates for intensified surveillance.

## Figures and Tables

**Table 1:** Odds ratios associated with truncating and missense CHEK2 mutations

**Site of cancer**	**Truncating mutation OR (95%CI)**	**Missense mutations OR (95% CI)**	**Reference**
Prostate	2.5 (1.6–3.7)	1.6 (1.3–2.1)	Cybulski *et al* (2004b)
Breast	3.3 (2.4–4.3)	1.6 (1.4–1.9)	Cybulski *et al* (2007a)
Colon	1.0 (0.5–1.8)	1.5 (1.2–2.0)	Cybulski *et al* (2007b)

CI=confidence interval; OR=odds ratio.

**Table 2:** Distribution of 431 cancers observed in first-degree relatives

**Cancer site**	**Number**
Prostate	68
Female genital	64
Breast	59
Colon	53
Stomach	38
Lung	33
Leukaemia	15
Liver	14
Pancreas	12
Kidney	10
Other	59
Total	431

Female genital includes ovary, uterine and cervical.

**Table 3:** Estimated cumulative risks for breast, prostate, colon and all cancers for first-degree relatives of patients with founder CHEK2 mutations

**Cancer site in relatives**	**CHEK2 mutation type**	**Cancer risk to age 50; same site in proband**	**Cancer risk to age of 50; all sites in proband**	**Cancer risk to age 75; same site in proband**	**Cancer risk to age of 75; all sites in proband**
Breast	Missense	0.032	0.014	0.108	0.053
	Truncating	0.069	0.035	0.091	0.076
	Either	0.042	0.019	0.104	0.059
Colon	Missense	0.015	0.005	0.075	0.035
	Truncating	0.000	0.000	0.046	0.020
	Either	0.012	0.003	0.070	0.031
Prostate	Missense	0.013	0.002	0.120	0.072
	Truncating	0.000	0.000	0.119	0.061
	Either	0.002	0.002	0.121	0.070
Any	Missense	—	0.039	—	0.216
	Truncating	—	0.045	—	0.248
	Either	—	0.041	—	0.223

**Table 4:** Risk factors for cancer in first-degree relatives of CHEK2 carriers and effects of mutations

	**HR**	**95% CI**	***P*-value**
*Breast cancer*			
Breast cancer in proband *versus* other	3.6	2.1–6.2	0.000004
Truncating *versus* missense mutation	1.4	0.8–2.5	0.2
*Prostate cancer*			
Prostate cancer in proband *versus* other	4.4	2.2–8.7	0.00002
Truncating *versu*s missense mutation	0.98	0.5–2.0	0.9
*Colon cancer*			
Colon cancer in proband *versus* other	4.2	2.4–7.8	0.000002
Truncating *versus* missense mutation	0.6	0.3–1.4	0.2

CI=confidence interval; HR=hazard ratio.

**Table 5:** Risk factors for prostate cancer in first-degree relatives of CHEK2 carriers

**Factor**	**HR**	**95% CI**	***P*-value**
Prostate cancer in proband *versus* other	4.4	2.2–8.7	0.00002
Truncating *versus* missense mutation	0.98	0.5–2.0	0.9

CI=confidence interval; HR=hazard ratio.

**Table 6:** Risk factors for colon cancer in first-degree relatives of CHEK2 carriers

**Factor**	**HR**	**95% CI**	***P*-value**
Colon cancer in proband *versus* other	4.2	2.4–7.8	0.000002
Truncating *versus* missense mutation	0.6	0.3–1.4	0.2

CI=confidence interval; HR=hazard ratio.
